# Disease‐related population declines in bats demonstrate non‐exchangeability in generalist predators

**DOI:** 10.1002/ece3.8978

**Published:** 2022-06-06

**Authors:** Amy K. Wray, Claudio Gratton, Michelle A. Jusino, Jing Jamie Wang, Jade M. Kochanski, Jonathan M. Palmer, Mark T. Banik, Daniel L. Lindner, M. Zachariah Peery

**Affiliations:** ^1^ 5228 Department of Forest & Wildlife Ecology University of Wisconsin‐Madison Madison Wisconsin USA; ^2^ 5228 Department of Entomology University of Wisconsin‐Madison Madison Wisconsin USA; ^3^ Center for Forest Mycology Research Northern Research Station USDA Forest Service Madison Wisconsin USA; ^4^ 5228 Department of Integrative Biology University of Wisconsin‐Madison Madison Wisconsin USA

**Keywords:** bat diet, community science, *Eptesicus fuscus*, high‐throughput sequencing, metabarcoding, *Myotis lucifugus*, white‐nose syndrome (WNS)

## Abstract

The extent to which persisting species may fill the functional role of extirpated or declining species has profound implications for the structure of biological communities and ecosystem functioning. In North America, arthropodivorous bats are threatened on a continent‐wide scale by the spread of white‐nose syndrome (WNS), a disease caused by the fungus *Pseudogymnoascus destructans*. We tested whether bat species that display lower mortality from this disease can partially fill the functional role of other bat species experiencing population declines. Specifically, we performed high‐throughput amplicon sequencing of guano from two generalist predators: the little brown bat (*Myotis lucifugus*) and big brown bat (*Eptesicus fuscus*). We then compared changes in prey consumption before versus after population declines related to WNS. Dietary niches contracted for both species after large and abrupt declines in little brown bats and smaller declines in big brown bats, but interspecific dietary overlap did not change. Furthermore, the incidence and taxonomic richness of agricultural pest taxa detected in diet samples decreased following bat population declines. Our results suggest that persisting generalist predators do not necessarily expand their dietary niches following population declines in other predators, providing further evidence that the functional roles of different generalist predators are ecologically distinct.

## Graphical abstract

While many bat species are known to function as suppressors of arthropod abundance, including several economically important agricultural pest taxa, the broader ecological consequences of disease‐related bat declines have not been fully assessed in many regions. In our study, we tested whether big brown bats (*Eptesicus fuscus*), which demonstrate lower mortality from white‐nose syndrome, could potentially fill the ecological role formerly occupied by the more severely affected little brown bat (*Myotis lucifugus*). Overall, we found that big brown bats did not adopt more of the prey formerly consumed by little brown bats and that the dietary niche overlap between the two bat species did not change over time.

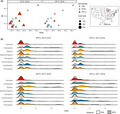

## 1 INTRODUCTION

Species tend to diverge and specialize over long periods of time, but generalist habits can be an asset in eras dominated by change. On an evolutionary scale, specialization can lead to speciation, yet the persistence of generalism may offer a buffer against extinction (Dennis et al., 2011; Loxdale et al., 2011). Flexible resource requirements are often considered advantageous in the rapidly changing environments of the Anthropocene (Boyles & Storm, 2007; Colles et al., 2009; Purvis et al., 2000). As certain species decline, the question of whether persisting sympatric species can serve as ecological replacements to maintain interaction networks becomes increasingly important (Parker et al., 2010; Rubenstein et al., 2006; Tylianakis et al., 2010). In some examples, such as the reintroduction of Galápagos tortoises with saddle‐backed phenotypes (*Chelonoidis* spp.) as replacements for saddle‐backed giant tortoises (*Chelonoidis abingdonii*, Hunter et al., 2013), intentional introductions of similar species with the purpose of restoration have been successful. Other examples, such as American mink (*Neogale vison*) populations increasing following the decline in red foxes (*Vulpes vulpes*, Carlsson et al., 2010) and grey squirrel (*Sciurus carolinensis*) populations increasing following the decline in red squirrels (*S*. *vulgaris*, Tompkins et al., 2003), demonstrate unintentional introductions or range expansions that involve one species adopting the vacant trophic niches left behind by another. Considering global predator declines and recent emphases on the role of predators in conservation biology (e.g., Estes et al., 2011; Ritchie et al., 2012), the question of whether and to what extent surviving predators may be capable of filling the functional roles of extirpated predators remains largely unanswered. Assessing the foraging flexibility of different taxa therefore represents an important step for developing conservation strategies focused on preserving ecosystem function in the era of species extinctions.

Whether extant persisting taxa can compensate for the functional roles of other declining populations is generally unknown in aerial arthropodivores, largely due to methodological challenges associated with characterizing the consumption of diverse prey communities prior to population declines. Anatomical and physiological constraints often limit the flexibility of generalist predator foraging. For example, among arthropodivorous bats, body size, flight agility and maneuverability, bite force, and echolocation call frequencies influence the type of prey that can be captured (Aguirre et al., 2003; Barclay & Brigham, 1991). Some bat species are capable of consuming prey larger than their own body size and will occasionally land and consume the preferred parts of the prey while discarding the rest (O'Shea & Vaughan, 1977; Santana & Cheung, 2016). Since most arthropodivorous bats rely on echolocation while hunting, certain types of echolocation are also adapted for specific prey and may play a role as a factor‐limiting dietary niche breadth and flexibility (Arbour et al., 2019). For example, while echolocation frequency is related to the habitats in which different bats forage, the diets of bats that use lower‐frequency echolocation are also more likely to include larger prey, while the diets of bats that use higher‐frequency echolocation are more likely to include smaller prey (Denzinger & Schnitzler, 2013; Jones & Holderied, 2007). Such physiological constraints are generally considered to be more important determinants of bat trophic niches than direct competition with other bats in similar guilds (Schoeman & Jacobs, 2011). As such, the morphological constraints that shape bat foraging strategies call into question the potential for ecological equivalency among taxa.

In North America, many hibernating bat species have experienced rapid and precipitous declines due to white‐nose syndrome (WNS), a disease caused by the fungus *Pseudogymnoascus destructans* (Frick et al., 2016; Lorch et al., 2011). Bat population declines from WNS present a unique circumstance under which basic ecological questions about the role of arthropodivorous bats as predators may be answered. In the eastern region of North America where WNS has been present for over a decade, little brown bats (*Myotis lucifugus*, Leconte 1831) have declined by more than 90% (Frick et al., 2016). Comparatively, other common bat species such as big brown bats (*Eptesicus fuscus*, Palisot de Beauvois 1796) experience infection from WNS yet have declined with much less severity (Frank et al., 2014; Frick et al., 2010). Additional studies have shown that some overlap in prey resource usage occurs between these two species in this study region, with molecular methods indicating a 21.3% overlap in OTUs detected in diets (Wray et al., 2021) and stable isotope methods indicating a 45% overlap between dietary profiles (Wray & Peery, 2022). These observed patterns, combined with the apparent lesser severity of big brown bat mortality due to WNS, raise the question of the extent to which persisting taxa may further expand their dietary profiles to include prey resources formerly consumed by other arthropodivorous bats that experience more severe population declines.

In this study, we quantified changes in dietary composition and niche overlap in two generalist arthropodivorous bat species following the rapid, WNS‐induced decline in one species (little brown bats) and the persistence of another (big brown bats). Specifically, we tested whether declines in little brown bats would lead to dietary niche expansion among big brown bats by comparing changes in prey consumption, including agricultural pests, as measured using high‐throughput amplicon sequencing methods.

## 2 METHODS

### 2.1 Study species

Little brown and big brown bats are among the most common bat species in North America (Fenton, 1980; Kurta & Baker, 1990). Little brown bats are higher‐frequency echolocators that tend to be generalist in their foraging habits, mostly consuming aquatic insects—particularly those with swarming behaviors (such as chironomid midges)—although they also consume terrestrial prey including moths, true bugs, beetles, and spiders (Clare, Symondson, Broders, et al., 2014; Whitaker & Lawhead, 1992; Wray et al., 2021). In contrast, big brown bats are lower‐frequency echolocators and are often speciously referred to as “beetle specialists” but are well known to consume a variety of other arthropods such as flies, caddisflies, true bugs, and moths (Agosta, 2002; Clare et al., 2014; Wray et al., 2021). We selected little brown bats as a focal study species because, prior to WNS‐related declines, they were the most abundant bat species in the study region (Huebschman, 2019). We selected big brown bats as a second focal study species because they are also abundant in the region but were expected to decline less from WNS (based on previous population trends observed in the eastern region of North America, e.g., Cheng et al., 2021), thus allowing for a comparative analysis of the effects of disease‐related bat declines in differentially afflicted species. During the breeding season, both little brown and big brown bats also frequently form large maternity roosts within human‐built structures (Voigt et al., 2016), which allows for ease of detection and monitoring between years.

### 2.2 Bat guano collection and detection of arthropod DNA

**FIGURE 1:**
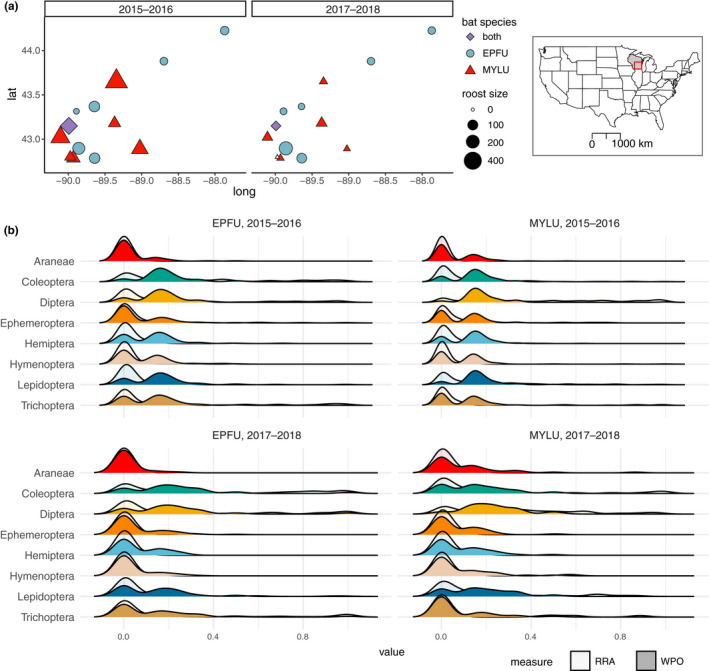
Characterization of study sites and bat dietary composition. (a) Map of study locations with points indicating the relative size of roosts. Map inset indicates the location of study sites within the continental United States. (b) Density plots of ordinal level dietary composition between time periods for each bat species. Solid regions represent weighted percent occurrence (wPO) values, and translucent regions indicate RRA (relative read abundance) values. EPFU = big brown bat (*Eptesicus fuscus*), MYLU = little brown bat (*Myotis lucifugus*)

We collected roost‐level bat guano samples (e.g., multiple pellets from multiple individuals at the same location) weekly at five little brown and five big brown bat roosts in Southern Wisconsin, USA, including one site with a little brown bat colony present in a bat house and a big brown bat colony present in a nearby barn (Figure 1a). We collected guano from each roost by placing a clean plastic sheet under each roost for 1 week, with samples collected weekly at all sites for the duration of the summer from 2015 to 2018 (late May to late August, ordinal weeks 20–36). Temporal differences in sample collection were minimized as much as possible, with a median ordinal collection week of 30 for the first 2 years (interquartile range [IQR] = 27, 32) and 29 for the second 2 years (IQR = 26, 32). Following collection, samples were stored on wet ice during transport and subsequently kept at –80°C for long‐term storage. Additional samples were collected in the same manner by community scientists at three or four time periods during the summer from 2015 to 2018, yielding an additional two little brown and two big brown bat roost sites (Figure 1a). These samples were initially stored at –20°C, then shipped overnight with a frozen ice pack and kept at –80°C for long‐term storage. This sample collection effort builds upon a previous study (Wray et al., 2021), with two additional years of data and five additional roost sites (*n *= 296 additional samples collected). The identity of bat species was confirmed by directly observing bat appearance (roosting bats were visible at all sites) and by comparing relative guano pellet size at the time of collection. The number of bats per roost was also estimated via emergence counts conducted as part of the Wisconsin Bat Program's Great Wisconsin Bat Count. These counts occurred at least twice per year in the early and late summer, which roughly corresponds to the pre‐ and post‐volancy reproductive periods. Approximately 30 minutes before sunset, volunteers positioned themselves near bat roosts and counted bats as they emerged. In 2015, little brown bat colonies had an average of 210 bats per roost (ranging from 94 to 409 bats), while big brown bat colonies had an average of 137 bats per roost (ranging from 21 to 428 bats). By 2018, little brown bat colonies declined to an average of 19 bats per roost (ranging from 0 to 50 bats), while big brown bat colonies declined to an average of 76 bats per roost (ranging from 26 to 180 bats). Overall, the average number of little brown bats in total per year was 1016 in the first 2 years, and 120 in the second 2 years, representing a decline of –88%. The average number of big brown bats in total per year was 929 in the first 2 years, and 542 in the second 2 years, representing a decline of –42%. All sample collection and animal observation methods were carried out in accordance with the guidelines of the Wisconsin Department of Natural Resources and the American Society of Mammalogists (Sikes, 2016). Experimental protocols were approved by the Wisconsin Natural History Inventory Program and the University of Wisconsin Animal Care and Use Committee.

### 2.3 Sequencing arthropod COI isolated from bat guano

DNA extraction, PCR, and high‐throughput amplicon sequencing followed Jusino et al. (2019) with the same modifications presented in Wray et al. (2021). Briefly, DNA was extracted from an 80mg subsample (~8 pellets) using a Qiagen DNA Stool mini kit (Qiagen Inc.), with a 180‐bp region of the COI subunit c amplified using PCR with ANML primers (Jusino et al., 2019). Thermocycler parameters followed Hebert et al. (2003), with the exception of the final extension at 72°C increased from 5 to 7 min. A single‐copy mock community of 34 known arthropod constituents was also amplified under the same conditions as a positive control (Jusino et al., 2019). Negative controls were included during each extraction batch and for each set of PCR reactions, which were visualized on a 2% agarose gel and did not yield any visible bands. Similarly, positive controls were also included for each set of PCR reactions, which did yield visible bands. PCR products were purified using a Zymo Select‐a‐Size Clean & Concentrator kit (Zymo Research), and then quantified using a Qubit 2.0 fluorometer (Invitrogen) with a high‐sensitivity dsDNA kit. Following quantification, the purified PCR products were then equilibrated. Five total equimolar libraries were then constructed, each with approximately 72 samples per library. Samples were processed in a randomized order to reduce potential batch processing biases. Sequencing was performed on an Ion Torrent Personal Genome Machine platform (PGM; ThermoFisher Scientific Inc.) according to the manufacturer's recommendations with an Ion PGM 318v2 chip. Raw sequence data were then processed using AMPtk v1.4.2 (Palmer et al., 2018). This data processing procedure includes de‐multiplexing using unique barcode index sequences, stripping of forward and reverse primers, and quality filtering and denoising with the DADA2 algorithm (Callahan et al., 2016). The resulting amplicon sequence variants (ASVs) were then clustered at 97% similarity using the UCLUST algorithm employed in VSEARCH to generate operational taxonomic units (OTUs; Jusino et al., 2019). Demultiplexed sequences were mapped back to these OTUs to generate an OTU table. Taxonomy was then assigned using the built‐in COI database in AMPtk. Finally, we removed all OTUs that were not identified as insects or arachnids, as well ectoparasites including mites (Mesostigmata and Trombidiformes), acari (Sarcoptiformes), and fleas (Siphonatera), which do not represent typical prey items.

### 2.4 Data cleaning and statistical analyses

Post‐processing, OTU tables were converted into weighted percent occurrence (wPO), a presence‐based metric, and relative read abundance (RRA), a read‐based metric, following Deagle et al. (2019). Since reads do not necessarily reflect abundance or biomass, occurrence‐based metrics are often considered more conservative, yet can be sensitive to overinflating the influence of rare taxa (Deagle et al., 2013, 2019). For this reason, we present both read‐based and occurrence‐based metrics for visual and qualitative comparison but use wPO for statistical analyses. Within major arthropod orders (Araneae, Coleoptera, Diptera, Ephemeroptera, Hemiptera, Hymenoptera, Lepidoptera, and Trichoptera), we compared mean values of wPO from samples collected between 2015 and 2016 with samples collected from 2017 to 2018 using separate Wilcoxon rank‐sum tests for each bat species. To account for the effects of multiple comparisons, we used a Bonferroni correction to adjust *p*‐values. We then aggregated OTU tables into presence/absence matrices at the family level for the estimation of niche metrics. We calculated niche breadth as Levin's adjusted niche breadth, *B_a_*, and calculated niche overlap as Pianka's measure of niche overlap, *O_jk_*, which provides a symmetrical estimate of the niche overlap between two species (Hurlbert, 1978; Levins, 1968; MacArthur & Levins, 1967). To visualize diet communities in multivariate space, we performed non‐metric multidimensional scaling (NMDS) using the metaMDS function in the R package “vegan” (Oksanen et al., 2013) with a modification of the raupcrick function as described by Chase et al. (2011), which was performed separately for presence/absence matrices at the OTU and family levels. For both taxonomic levels, we excluded any taxon groups that were not detected at least five times in order to reduce the influences of infrequently detected diet items. To focus on general trends in community composition, outliers were also sequentially removed following a visual inspection of NMDS plots (*n *= 12 samples removed). To assess whether the bat diet communities differed by species, time period, collection site, and ordinal week, we used non‐parametric PERMANOVA tests (Anderson, 2001) that were performed by the “adonis” function with 999 replicates and assessed the influences of multivariate dispersion (Anderson, 2006) using the “betadisper” function to separately test each predictor variable. We also searched taxonomy tables for certain arthropod taxa that are known agricultural pests in the area. To compare the incidence and taxonomic richness of pest taxa detected in samples collected between 2015 and 2016 with samples collected between 2017 and 2018, we used chi‐squared tests and Welch's *t*‐tests, respectively, which were conducted separately for each bat species. All post‐OTU table data cleaning and analyses were conducted in R version 4.1.0 (R Core Team, 2021). Additional packages used for data processing and visualization include “dplyr”, “ggplot2”, “tidyverse”, “wesanderson”, and “reshape2” (Ram & Wickham, 2018; Wickham, 2007, 2016; Wickham et al., 2019).

## 3 RESULTS

A total of 350 samples were successfully amplified of 558 samples collected (62.7%), yielding 20,990,143 sequencing reads. Following filtering and bioinformatic processing, 326 of the amplified samples (93.1%) were retained, which yielded a total of 2274 valid OTUs with 16,777,947 sequencing reads. All members of the insect mock community were recovered. After excluding sample duplicates (*n *= 11), the final sample set included 173 little brown and 142 big brown bat samples (*n *= 315 total samples). All OTUs were identified to phylum, class, and order, with 87.4% identified to family, 74.8% identified to genus, and 56.9% identified to species. After removing non‐arthropod OTUs and taxa that were unlikely prey items (i.e., ectoparasites, *n *= 611 OTUs removed), 1663 arthropod prey OTUs remained (73% of the total valid OTUs), representing 19 orders, 221 families, 703 genera, and 891 species. Samples had a median of 41,666 reads per sample (IQR = 22,080–54,122) and a median of 17 OTUs per sample (IQR = 9–27). A total of 1334 OTUs were found among little brown bats and 865 OTUs were found among big brown bats, of which 536 OTUs were shared between both. The most detected prey families for little brown bats, as measured by incidence, were Diptera: Chironomidae, followed by Lepidoptera: Tortricidae and Diptera: Limoniidae. For big brown bats, the most detected prey families were Coleoptera: Elateridae, Diptera: Limoniidae, and unidentified Coleoptera (Table 1). The most common prey, as measured by wPO, and relative reads were generally consistent, except for big brown bats where Trichoptera: Hydropsychidae had a much higher RRA in comparison to wPO (Table 1).

**TABLE 1:** Twenty most commonly detected prey families for each bat species, ranked by incidence

Little brown bat (*Myotis lucifugus*)					Big brown bat (*Eptesicus fuscus*)				
Order	Family	Incidence	Mean RRA	Mean wPO	Order	Family	Incidence	Mean RRA	Mean wPO
Diptera	Chironomidae	138	0.213	0.077	Coleoptera	Elateridae	83	0.160	0.061
Lepidoptera	Tortricidae	93	0.037	0.041	Diptera	Limoniidae	71	0.100	0.055
Diptera	Limoniidae	78	0.036	0.031	Coleoptera	unidentified	70	0.010	0.045
Coleoptera	Elateridae	75	0.057	0.036	Coleoptera	Carabidae	67	0.018	0.047
Diptera	unidentified	71	0.013	0.028	Trichoptera	Hydropsychidae	65	0.196	0.070
Lepidoptera	Gelechiidae	58	0.015	0.028	Coleoptera	Scarabaeidae	56	0.075	0.049
Ephemeroptera	Caenidae	53	0.044	0.022	Lepidoptera	Tortricidae	54	0.030	0.037
Hemiptera	Miridae	51	0.011	0.022	Diptera	Chironomidae	45	0.063	0.036
Lepidoptera	Depressariidae	50	0.049	0.020	Coleoptera	Pyrochroidae	43	0.020	0.028
Trichoptera	Hydropsychidae	49	0.083	0.028	Diptera	Tipulidae	42	0.004	0.023
Diptera	Culicidae	46	0.028	0.021	Coleoptera	Cerambycidae	36	0.007	0.022
Diptera	Tipulidae	44	0.003	0.019	Hemiptera	Miridae	35	0.010	0.020
Hemiptera	Corixidae	41	0.021	0.017	Coleoptera	Hydrophilidae	33	0.026	0.021
Trichoptera	Leptoceridae	40	0.016	0.017	Ephemeroptera	Heptageniidae	32	0.044	0.025
Ephemeroptera	Heptageniidae	39	0.017	0.018	Megaloptera	Corydalidae	30	0.058	0.029
Diptera	Psychodidae	37	0.028	0.015	Hymenoptera	Ichneumonidae	27	0.004	0.015
Hymenoptera	Ichneumonidae	34	0.002	0.013	Diptera	unidentified	26	0.005	0.022
Coleoptera	unidentified	32	0.002	0.014	Coleoptera	Tenebrionidae	21	0.003	0.013
Coleoptera	Carabidae	31	0.013	0.015	Hemiptera	Cicadellidae	21	0.000	0.013
Diptera	Ceratopogonidae	30	0.001	0.010	Coleoptera	Silphidae	21	0.001	0.010

Abbreviations: RRA, relative read abundance; wPO, weighted percent occurrence.

Overall, we found that little brown and big brown bat dietary composition was distinct, and intraspecific differences in dietary composition did not change substantially between pre‐ and post‐WNS time periods for either bat species. Little brown bats diets contained a higher richness of Diptera, Lepidoptera, and Hemiptera, and big brown bat diets contained a higher richness of Coleoptera. The mean wPO within arthropod orders differed by bat species but remained similar between time periods within each bat species (Figure 1b; Table 2). Hemiptera were less common in both little brown and big brown bat guano samples in the later time periods, and for little brown bats, other prey groups did not change (Figure 1b; Table 2). In the later time period, Hymenoptera were marginally less common in big brown bat guano samples (Figure 1b; Table 2). The arthropod families most commonly detected for each bat species also remained similar between time periods (Table 3).

**TABLE 2:** Changes in mean weighted percent occurrence (wPO) of prey orders between time periods

Order	Big brown bat (*Eptesicus fuscus*)				Little brown bat (*Myotis lucifugus*)			
	Mean, 2015–2016	Mean, 2017–2018	*p*	*p*, adjusted	Mean, 2015–2016	Mean, 2017–2018	*p*	*p*, adjusted
Araneae	0.033	0.020	.229	1.000	0.059	0.091	.341	1.000
Coleoptera	0.180	0.231	.102	.814	0.141	0.153	.991	1.000
Diptera	0.155	0.220	.037	.295	0.185	0.222	.032	.260
Ephemeroptera	0.067	0.050	.339	1.000	0.088	0.058	.067	.537
**Hemiptera**	**0.118**	**0.067**	.**004**	.**035**	**0.124**	**0.079**	.**005**	.**041**
Hymenoptera	0.078	0.041	.007	.056	0.084	0.067	.098	.786
Lepidoptera	0.147	0.129	.370	1.000	0.159	0.172	.828	1.000
Trichoptera	0.122	0.136	.636	1.000	0.092	0.069	.082	.657

Note

Adjusted *p*‐values below .05 are highlighted in bold.

**TABLE 3:** Top 20 family‐level prey items detected in big brown and little brown bat guano samples, ranked by weighted percent occurrence (wPO). Changes in top family‐level prey items between time periods are highlighted in bold

Big brown bat (*Eptesicus fuscus*), 2015–2016			Big brown bat (*Eptesicus fuscus*), 2017–2018		
Order	Family	Mean wPO	Order	Family	Mean wPO
Coleoptera	Elateridae	0.0527	Trichoptera	Hydropsychidae	0.089
Trichoptera	Hydropsychidae	0.0524	Coleoptera	Scarabaeidae	0.070
Coleoptera	Carabidae	0.0498	Coleoptera	Elateridae	0.070
Coleoptera	unidentified	0.0469	Diptera	Limoniidae	0.064
Diptera	Limoniidae	0.0459	Coleoptera	Carabidae	0.044
Lepidoptera	Tortricidae	0.0376	Coleoptera	unidentified	0.044
Ephemeroptera	Heptageniidae	0.0366	Megaloptera	Corydalidae	0.043
Diptera	Chironomidae	0.0330	Diptera	Chironomidae	0.040
Coleoptera	Scarabaeidae	0.0305	Lepidoptera	Tortricidae	0.036
Coleoptera	Pyrochroidae	0.0269	Coleoptera	Pyrochroidae	0.030
Coleoptera	Cerambycidae	0.0262	Diptera	unidentified	0.024
Hemiptera	Miridae	0.0241	Diptera	Tipulidae	0.023
Diptera	Tipulidae	0.0222	Coleoptera	Hydrophilidae	0.021
Coleoptera	Hydrophilidae	0.0213	**Hemiptera**	**Cicadellidae**	**0.019**
Diptera	unidentified	0.0196	Coleoptera	Cerambycidae	0.017
Hymenoptera	Ichneumonidae	0.0180	Hemiptera	Miridae	0.015
Megaloptera	Corydalidae	0.0167	**Diptera**	**Culicidae**	0.014
**Lepidoptera**	**Tineidae**	**0.0148**	Coleoptera	Tenebrionidae	0.014
**Trichoptera**	**Leptoceridae**	**0.0135**	Ephemeroptera	Heptageniidae	0.012
Coleoptera	Tenebrionidae	0.0128	Hymenoptera	Ichneumonidae	0.012
Little brown bat (*Myotis lucifugus*), 2015–2016			Little brown bat (*Myotis lucifugus*), 2017–2018		
Diptera	Chironomidae	0.0744	Diptera	Chironomidae	0.086
Lepidoptera	Tortricidae	0.0387	Lepidoptera	Tortricidae	0.049
Diptera	Limoniidae	0.0337	Coleoptera	Elateridae	0.044
Coleoptera	Elateridae	0.0337	Trichoptera	Hydropsychidae	0.043
Diptera	unidentified	0.0285	Diptera	Culicidae	0.032
Lepidoptera	Gelechiidae	0.0268	Lepidoptera	Gelechiidae	0.031
**Ephemeroptera**	**Caenidae**	**0.0259**	Lepidoptera	Depressariidae	0.031
**Hemiptera**	**Miridae**	**0.0251**	Diptera	unidentified	0.028
Trichoptera	Hydropsychidae	0.0240	**Araneae**	**unidentified**	**0.025**
**Trichoptera**	**Leptoceridae**	**0.0207**	**Coleoptera**	**Scarabaeidae**	**0.025**
**Hemiptera**	**Corixidae**	**0.0193**	**Coleoptera**	**Dermestidae**	**0.024**
Diptera	Tipulidae	0.0184	Diptera	Tipulidae	0.023
Ephemeroptera	Heptageniidae	0.0182	**Araneae**	**Theridiidae**	**0.021**
Lepidoptera	Depressariidae	0.0176	**Coleoptera**	**unidentified**	**0.021**
Diptera	Culicidae	0.0175	Diptera	Limoniidae	0.019
**Diptera**	**Psychodidae**	**0.0166**	**Diptera**	**Chaoboridae**	**0.019**
**Hymenoptera**	**Ichneumonidae**	**0.0155**	Ephemeroptera	Heptageniidae	0.018
**Diptera**	**Cecidomyiidae**	**0.0154**	Coleoptera	Carabidae	0.018
**Lepidoptera**	**Tineidae**	**0.0148**	**Diptera**	**Tachinidae**	**0.017**
Coleoptera	Carabidae	0.0140	**Lepidoptera**	**Crambidae**	**0.015**

**FIGURE 2:**
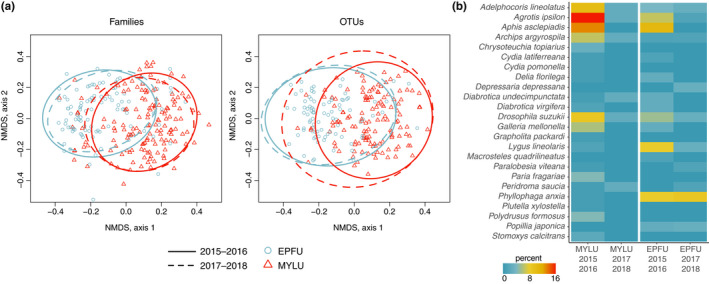
Changes in bat dietary composition over time. (a) NMDS plot of family‐level and OTU‐level dietary communities with 80% confidence interval ellipses. Solid lines indicate the first time period (2015–2016), while dashed lines indicate the second time period (2017–2018). Shapes indicate samples from each bat species. (b) Heatmap of agricultural pest taxa detected in bat guano samples. Values indicate the percentage of samples for which each agricultural pest taxa were detected. EPFU = big brown bat (*Eptesicus fuscus*), MYLU = little brown bat (*Myotis lucifugus*)

In general, little brown and big brown bats showed interspecific differences in family‐level dietary niche breadth, which did not change substantially between the pre‐ and post‐WNS time periods. Little brown bats displayed higher niche breadth in comparison to big brown bats, and total interspecific niche overlap was 0.281 (Table 4). For both bat species, dietary niche breadth from samples collected in 2015–2016 was higher than dietary niche breadth from samples collected in 2017–2018. Little brown bat dietary niche breadth decreased by 24.2% between time periods, while big brown bat dietary niche breadth decreased by 34.6% between time periods (Table 4). Interspecific dietary niche overlap was similar between time periods, increasing by only 2.3% from 0.281 to 0.288 (Table 4). NMDS plots indicated visually that diet composition differed more between species than between time periods (Figure 2a). PERMANOVA demonstrated that species was the best predictor of variation at the family and OTU levels (Figure 2a; Table 5). At both the family and OTU levels, ordinal week was also a significant predictor of variation, while collection site and time period were also significant predictors at the OTU level (Table 5). At both the family and OTU levels, there were significant differences in multivariate dispersion between bat species and between collection sites, and at the OTU level, there were also significant differences in multivariate dispersion between time periods and ordinal week (Figure 2a; Table 5). These results suggest that while bat species was the most important factor in determining diet community structure and dispersion, other factors were also somewhat influential, particularly at the OTU level.

**TABLE 4:** Family‐level dietary niche breadth (*B*, Levin's measure of niche breadth and *B_a_*, Levin's adjusted niche breadth) and overlap *O_jk_* (Pianka's measure of symmetrical niche overlap) for little brown and big brown bats

Time	*B*, MYLU	*B_a_*, MYLU	*B*, EPFU	*B_a_*, EPFU	Overlap, *O_jk_*
2015–2018	51.3252	0.2207	35.8268	0.1527	0.2813
2015–2016	52.1028	0.2241	42.0538	0.1801	0.2811
2017–2018	39.7123	0.1698	27.8405	0.1177	0.2876

Abbreviations: EPFU, big brown bat (*Eptesicus fuscus*);MYLU, little brown bat (*Myotis lucifugus*).

**TABLE 5:** PERMANOVA and Betadisper test results

Term	PERMANOVA						Betadisper	
	df	Sums of Sqs	Mean Sqs	*F*	*R* ^2^	*p*	*F*	*p*
Family level								
**Species**	**1**	**2.42**	**2.416**	**13.97**	0.**052**	.**01**	**15.89**	**<.001**
Site	12	2.83	0.236	1.36	0.061	.14	**1.89**	.**04**
Time	1	0.29	0.286	1.65	0.006	.24	0.01	.92
**Week**	**1**	**0.68**	**0.682**	**3.94**	0.**015**	.**03**	0.99	.47
Residuals	233	40.30	0.173		0.866			
Total	248	46.52			1.000			
OTU level								
**Species**	**1**	**10.12**	**10.124**	**62.14**	0.**117**	.**01**	**16.40**	**<.001**
**Site**	**13**	**32.25**	**2.481**	**15.22**	0.**371**	.**01**	**3.17**	**<.001**
**Time**	**1**	**2.20**	**2.202**	**13.51**	0.**025**	.**01**	**3.98**	.**047**
**Week**	**1**	**2.00**	**2.004**	**12.30**	0.**023**	.**01**	**2.90**	**<.001**
Residuals	247	40.25	0.163		0.464			
Total	263	86.82			1.000			

Note

*P* values below .05 are highlighted in bold.

Several agricultural pests were detected in bat guano samples, which were most common in little brown bat samples collected prior to WNS‐related declines. Cumulatively, we detected at least one agricultural pest taxon in 45.1% of little brown bat samples and in 33.8% of big brown samples. For little brown bats, this percentage decreased from 53.3% to 15.8% between the two time periods, while for big brown bats this percentage decreased from 43.2% to 23.6% between the two time periods. The most common agricultural pest taxon in little brown bat guano samples was *Agrotis ipsilon* (black cutworm), which was detected in 13% of all samples, while the most common agricultural pest taxon for big brown bats was *Phyllophaga anxia* (cranberry white grub), which was detected in 8% of all samples. For both bat species, the incidence of most individual pest taxa also declined between time periods (Figure 2b). The difference in the proportion of samples with at least one agricultural pest taxon present between time periods was statistically significant for little brown bats (*χ* ^2^ = 15.4, *df* = 1, *p *< .001) and for big brown bats (*χ* ^2^ = 5.31, *df* = 1, *p* = .021). Little brown bats had an average of 0.54 pest taxa per sample (ranging from 0 to 4 pest taxa per sample), while big brown bats had an average of 0.45 pest taxa per sample (ranging from 0 to 3 pest taxa per sample). For little brown bat guano samples, the average richness of pest taxa per sample was significantly higher in the first time period (*x̅* = 0.89 ± 0.17) in comparison to the second time period (*x̅* = 0.18 ± 0.15, *t*_136.61_ = 6.19, *p *< .001). For big brown bat guano samples, the average richness of pest taxa per sample was also significantly higher in the first time period (*x̅* = 0.61 ± 0.19) in comparison to the second time period (*x̅* = 0.29 ± 0.15, *t*_133.18_ = 2.61, *p *= .01).

## 4 DISCUSSION

Overall, our findings indicate that big brown bats do not immediately expand their dietary profiles after rapid declines in little brown bat populations, and are therefore unlikely to function as ecological replacements. In this study, we tested whether the effects of bat population declines influenced changes in dietary composition, interspecific niche overlap, and the amount of agricultural pest taxa consumed. Little brown bat roost sizes declined to a much greater extent than big brown bats, in accordance with our *a priori* assumptions. After the observed declines in little brown bat populations, intraspecific dietary composition did not change substantially, nor did overlap increase, although niche breadth decreased for both bat species. These results indicate that while little brown and big brown bats provide some complementarity as predators with different dietary niches, they already maintained some dietary niche overlap prior to WNS‐related population declines, which is also consistent with a long‐term study using stable isotope analyses (Wray & Peery, 2022). The observed decrease in niche breath following declines in roost sizes suggests that both bat species may display some degree of individual specialization (Bolnick et al., 2002, 2003), with the individuals within a population consuming different prey resources that ultimately contribute to a broader population‐level dietary niche. The decreasing prevalence of agricultural pest arthropods in bat diets following WNS‐related declines may be an artefact of overall decreasing dietary niche breadth but could also suggest that individuals select for other prey items, potentially as a result of reduced inter‐ or intraspecific competition. These results are consistent with one previous study which also suggested that big brown bat dietary niche breadth may be driven by individual specialization (Cryan et al., 2012), although the contributions of individuals to population‐level dietary breadth have been seldom explored in arthropodivorous bats despite evidence of within‐population variation in several other taxa (Johnston & Fenton, 2001).

While niche breadth decreased for both bat species, niche overlap did not change, but rather, some niche overlap (0.281) was observed prior to declines in bat populations. One previous study, which compared pre‐ and post‐WNS dietary composition based on stomach contents of bat carcasses, found that overlap in dietary composition did increase following WNS‐related bat declines and suggested that this may indicate increasing competition (Morningstar et al., 2019). Considering the observed variation between sites and between weeks, as well as the known high spatial and temporal turnover in the diets of both bat species (Wray et al., 2021), the comparison of pre‐ and post‐WNS bat diets may not be appropriate for individual bats collected at different sites or during different seasonal time periods. Interannual variation in diets may also influence conclusions for studies with limited or uneven year‐to‐year sampling schemes. Separate studies also suggested that pre‐ and post‐WNS changes in bat acoustic activity could indicate shifts in temporal and spatial niche partitioning (Jachowski et al., 2014) or reduced interspecific competition (Mayberry et al., 2020). However, just as co‐occurrence does not necessarily imply the presence of ecological interactions (Blanchet et al., 2020), decreasing habitat or dietary niche overlap does not necessarily imply decreasing competition. The principle of competition relies on the supposition that resources shared by two species must be limiting for competition to occur, and coexistence has been shown to persist under many cases where the assumptions of competitive exclusion are not met (Chase et al., 2002; Holt, 1977). As such, the results of our study suggest that WNS‐related declines in little brown bats likely do not lead to increases in niche overlap in this study area, but rather demonstrates that interspecific niche overlap between little brown and big brown bats has remained fairly consistent in recent time.

The results of this study suggest that while big brown bats share some prey resources with little brown bats, they do not readily shift their diets to include more prey resources following little brown bat declines. These findings are consistent with previous research and suggest that big brown bat foraging may be limited by other factors such as body size. Indeed, little brown and big brown bats are estimated to have diverged from each other more than 30 million years ago (Amador et al., 2018; Lack & Bussche, 2010), and have developed adaptations for foraging on different prey types. In this study, we did not quantify the influences of prey availability, although other studies have detected declining arthropod abundance in many regions (Hallmann et al., 2017; Sánchez‐Bayo & Wyckhuys, 2019; Seibold et al., 2019). A previous study in this region showed that these bat species generally maintain strong prey preferences independently of changing local prey availability (Wray et al., 2021). Nonetheless, other factors, such as habitat or roost availability, could represent limiting resources where the larger body size of big brown bats could make for a better competitor (Agosta, 2002). We observed complete roost abandonment at two little brown bat roost sites by 2018, and if these roosts were later adopted by big brown bats, it may be unlikely that little brown bats could reoccupy them upon population recovery. As such, further exploration into the potential competition between little brown and big brown bats for roost space or other habitat requirements may be warranted and are potentially more important than limitations due to food resources.

We characterized the functional role of bats as predators with the goal of assessing the extent to which widespread, flexible, and comparatively successful sympatric species have the potential to serve as ecological replacements for other declining species. While big brown bats likely cannot fully fill the trophic role of little brown bats, possibly due to morphological or other physiological constraints, other bat species may be more ecologically similar to little brown bats. However, most of these species also experience severe declines due to WNS, and non‐affected species such as migratory bats do not cluster in large colonies in this study region (Huebschman, 2019), and as such probably do not influence prey communities in the same manner. While other studies have demonstrated the successful reintroductions of extirpated predators leading to restoration ecosystem functioning (e.g., Mittelbach et al., 1995; Ripple & Beschta, 2012), such efforts often rely on the possibility of conservation strategies such as captive breeding or translocation—none of which have successfully been implemented for little brown bats or other bat species severely affected by WNS (Davy & Whitear, 2016). As such, it is unlikely that the functional role of the little brown bat can be restored either naturally or through management strategies, and several bat species severely impacted by WNS are expected to face extirpation in many regions (Frick et al., 2010; Thogmartin et al., 2013). The growing body of evidence regarding the function of arthropodivorous bats as ecologically important predators thus raises serious concerns regarding potential top‐down consequences of WNS‐related bat declines. These findings highlight the importance of continuing to support little brown bat population recovery, while also emphasizing the need for conservation of bats and other aerial arthropodivores in general due to the probability that each unique species cannot necessarily be replaced by another.

## AUTHOR CONTRIBUTIONS

**Amy Wray:** Data curation (lead); Formal analysis (lead); Investigation (lead); Visualization (lead); Writing—original draft (lead); Writing—review & editing (lead). **Claudio Gratton:** Conceptualization (equal); Funding acquisition (equal); Writing—review & editing (supporting). **Michelle Jusino:** Data curation (supporting); Investigation (supporting); Methodology (lead); Resources (supporting); Writing—review & editing (supporting). **Jing Jamie Wang:** Investigation (supporting); Writing—review & editing (supporting). **Jade Kochanski:** Investigation (supporting); Writing—review & editing (supporting). **Jonathan M Palmer:** Data curation (supporting); Methodology (supporting); Software (supporting); Writing—review & editing (supporting). **Mark Banik:** Methodology (supporting); Resources (supporting); Writing—review & editing (supporting). **Daniel L. Lindner:** Conceptualization (equal); Resources (supporting); Writing—review & editing (supporting). **Zach Peery:** Conceptualization (equal); Funding acquisition (equal); Resources (lead); Writing—review & editing (supporting).

## CONFLICT OF INTEREST

The authors declare no conflicts of interest.

## Data Availability

The final OTU table and sample metadata are permanently archived online and can be accessed at https://figshare.com/articles/dataset/ch4_OTU_table_taxonomy_with_metadata/17267150. Raw sequence data are available at SRA accession number PRJNA668526.
